# Molecular Characterization of Lineage-IV Peste Des Petits Ruminants Virus and the Development of In-House Indirect Enzyme-Linked Immunosorbent Assay (IELISA) for its Rapid Detection”

**DOI:** 10.1186/s12575-024-00249-y

**Published:** 2024-07-05

**Authors:** Tahira Kamal, Saeed-ul-Hassan Khan, Fariha Hassan, Amir-bin- Zahoor, Amman Ullah, S. Murtaza Hassan Andrabi, Ghulam Muhammad Ali, Tayyaba Afsar, Fohad Mabood Husain, Huma Shafique, Suhail Razak

**Affiliations:** 1 National Institute for Genomics and Advanced Biotechnology, National Agricultural Research Center, Islamabad, Pakistan; 2 Animal Sciences Institute, Animal Health, National Agricultural Research Center, Islamabad, Pakistan; 3 Department of Microbiology, Faculty of Biological Sciences, Quaid-i-Azam University, Islamabad, Pakistan; 4 Department of Zoology, Faculty of Biological Sciences, Quaid-i-Azam University, Islamabad, Pakistan; 5 Department of Community Health Sciences, College of Applied Medical Sciences, King Saud University, Riyadh, Saudi Arabia; 6 Department of Food Science and Nutrition, College of Food and Agriculture Sciences, King Saud University, Riyadh, Saudi Arabia; 7 Institute of Cellular Medicine, Newcastle University Medical School, Newcastle University, Upon Tyne, UK

**Keywords:** IELISA, PPRV, RT-PCR, Cost-effective, Diagnostic assays

## Abstract

Peste des petits ruminants (PPRV), a highly contagious viral disease, causes significant economic losses concerning sheep and goats. Recently, PPR viruses (PPRVs), have adopted new hosts and lineage IV of PPRVs represents genetic diversity within the same lineage. 350 samples, including blood, swabs, and tissues from sheep/goats, were collected during the 2020–2021 disease outbreaks in Pakistan. These samples were analysed through RT-PCR and three isolates of PPRV with accession numbers, MW600920, MW600921, and MW600922, were submitted to GenBank, based on the partial N-gene sequencing. This analysis provides a better understanding of genetic characterizations and a targeted RT-PCR approach for rapid PPRV diagnosis. An IELISA test was developed using the semi-purified antigen MW600922 isolate grown in Vero cells. The PPRV isolates currently present high divergence with the Turkish strain; conversely, similarities equivalent to 99.73% were observed for isolates collected from Pakistan. The developed indirect ELISA (IELISA) test demonstrated antibody detection rates at dilutions of 1:200 for antibodies (serum) and 1:32 for antigens. In comparison to cELISA, high specificity (85.23%) and sensitivity (90.60%) rates were observed. In contrast to the virus neutralization test (VNT), IELISA was observed to be 100% specific and 82.14% sensitive in its results. Based on these results, serological surveys conducted for PPR antibodies using IELISA can be a more effective strategy on a larger scale. Furthermore, our results demonstrate a significant breakthrough in the research in terms of cost-effectiveness and storage efficiency, and the developed IELISA test is highly recommended for use in developing countries.

**Supplementary Information:**

The online version contains supplementary material available at 10.1186/s12575-024-00249-y.

## Abstract (plain language summary)

Peste des petits ruminants (PPRV) is a transboundary, highly contagious, and economically significant viral disease affecting small ruminants and wildlife. PPRV, a disease that only targets animals, is the focus of the Global Eradication Programme (PPRV GEP), which aims to eradicate the disease by 2030. Following the completion of the first phase of the GEP (2017–2021), Pakistan has initiated the second phase: PPRV presence and the implementation of a control strategy. Rapid and accurate laboratory diagnosis is vital to the disease’s effective control and eradication. In the present study, we have improved diagnosis by reverse transcriptase polymerase chain reaction (RT-PCR), which not only can detect low viral concentrations but also contributes to the genetic analysis of lineage-IV viruses. However, the development of cost-effective indirect ELISA (iELISA) may allow for the analysis of serum samples obtained from larger populations of small ruminants.

**Supplementary Information:**

The online version contains supplementary material available at 10.1186/s12575-024-00249-y.

## Background

The acute, highly contagious viral disease goat plague also known as peste des petits ruminants (PPRV), results in significant economic losses because of its high morbidity and mortality rate [1, 2]. Primarily affecting sheep and goats, this disease can also occasionally infect camels and wild small ruminants [3, 4]. PPRV is caused by the Small Ruminant Morbillivirus, which belongs to the Paramyxoviridae family. The PPR virus is enclosed within a capsid having a genome of single-stranded RNA. The PPRV is considered as the biggest member of the *Small ruminant Morbillivirus* genus [5]. The PPR virus’s genome has a total of six structural encoded proteins by a matrix protein (M), fusion protein (F), hemagglutinin protein (H) nucleoprotein (N), RNA-dependent polymerase (L) and RNA-polymerase phosphoprotein co-factor (P). Based on the PPR virus strains’ (N, F, or H genes) sequence analysis, the N gene was found to be best for molecular characterization of isolates [6].

Previous PPRV studies, based on genetic genotyping, revealed four lineages [7, 8], each lineage has a distinct geographic distribution pattern that has altered recently. Lineage IV is found in the Arabian Peninsula, the Middle East, and South Asia, while Lineage III is found in Eastern Africa and some regions of the Middle East. Similarly, the Lineage II isolates are found in Western Africa whereas the Lineage I is mainly found in West Africa and has been reported to exist in Central Africa. According to recent studies, lineage IV of the PPR virus has emerged in Pakistan and the surrounding countries [9]. Pakistan is a country in South Asia where FAO (Food and Agriculture Organization) plans to eradicate PPR by 2030 [10]. Pakistan loses PKR 20.5 billion (USD 0.24 billion) annually due to PPRV cases [11]. Pakistan has over 8000 outbreaks documented between 2005 and 2018. These outbreaks of PPRV are highly damaging to the economy; inflicting varying socio-economic impacts around the year [12].

Although the unique geographical dissemination of the virus based on genotyping indicates that it existed long before it was discovered in the places indicated, subsequent incidence of unexpected lineage of virus that was introduce in the regions previously linked to other lineages reveal that the virus was moving [13]. Moreover, recent cases of PPRV in Pakistan show the mobility of PPRV in this region shows the mobility of PPRV and its dynamic epidemiological state [9, 14].

The PPR can readily be detected in various clinical specimens obtained from infected sheep and goats, including skin nodules, ulcerations, semen, blood, and serum [15]. Detection methods encompass polymerase chain reaction (RT-PCR), Serology, and virus isolation and culturing, with RT-PCR being the most commonly employed due to its cost-effectiveness, simplicity, and high sensitivity and specificity, notwithstanding the utilization of live attenuated vaccination [11, 16].

Despite Pakistani publications predominantly focusing on clinical or serology results post the 1995 PPRV outbreak, only one study included molecular characterization and phylogenetic analysis based on the F gene [17]. Establishing the characteristics of circulating PPR virus strains is crucial for advancing diagnostic and control strategies [18]. Considering these previous studies, our studies focused on identifying PPR viruses based on N-gene to improve their molecular diagnosis. Three PPRV virus isolates were then partially sequenced using the N-gene for lineage identification purposes. These sequences have been submitted to NCBI. We also aimed to determine the phylogenetic relation of PPRV to identify circulating viruses. Therefore, we used indigenous PPRV isolate isolated from tissue samples for the development of an in-house ELISA (IELISA) assay. The effectiveness of the newly developed IELISA was then evaluated by comparing it to a commercial cELISA kit. Additionally, the phylogenic analysis we performed will aid in our understanding of disease spread. In Pakistan, small ruminant outbreaks continue to occur, necessitating further research into the molecular details of circulating field viruses.

## Methods

### Sample Collection

**Fig. 1:**
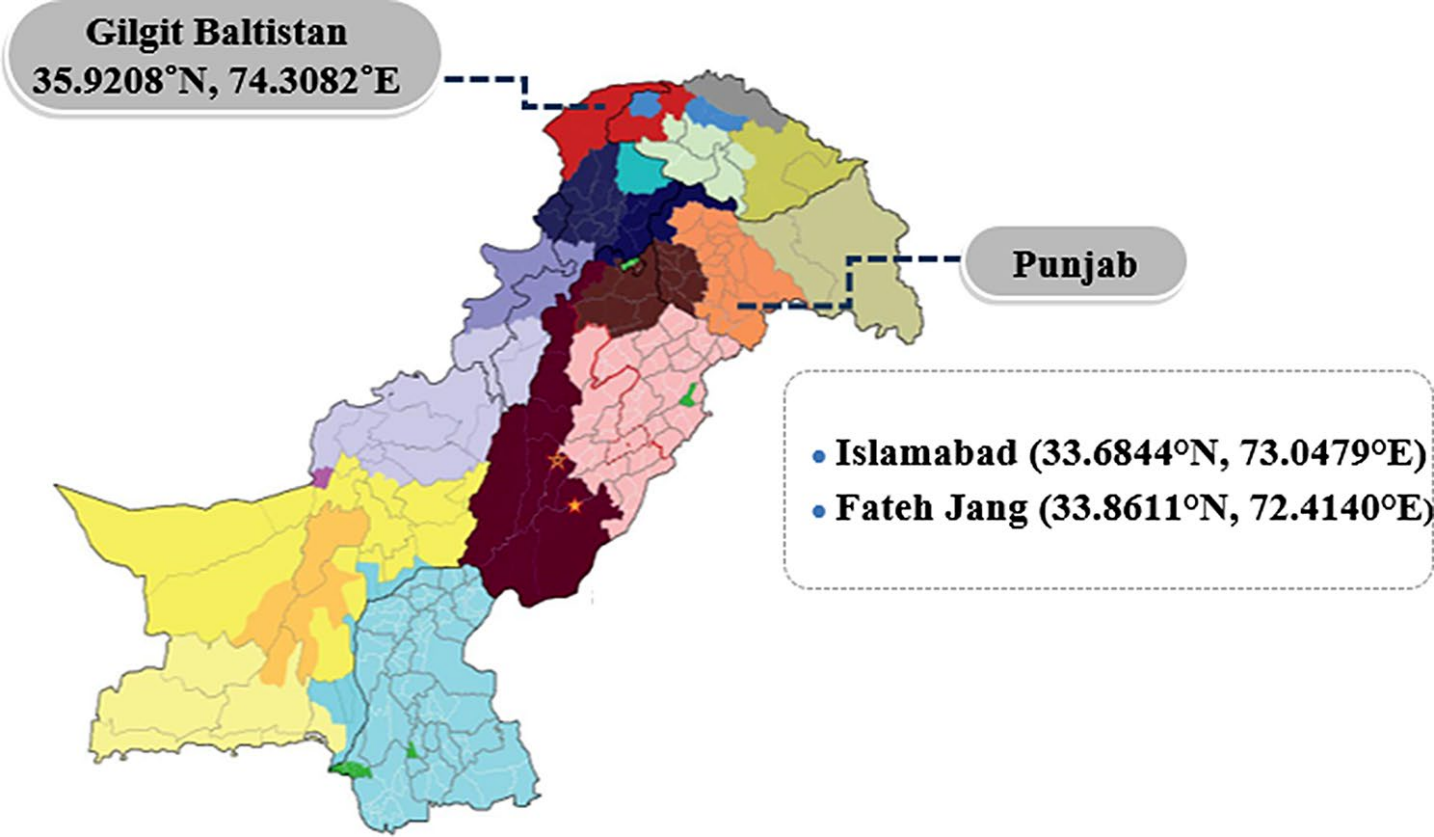
Locations of serum sample collections for sheep and goats during active outbreaks of PPRV reported from Pakistan, in 2020–2021 (colours indicate different divisions under provinces)

During 2020–2021, the suspected outbreaks of PPR were reported by field veterinarians, within three different regions including Islamabad territory (33.6844° N, 73.0479°), Fateh Jang (33.8611° N, 72.4140° E), and Gilgit (35.9208° N, 74.3082°E). These animals were not vaccinated ever (Fig. 1). A total of 325 blood samples, including 120 sheep and 205 goats were randomly collected from apparently healthy as well as having typical PPRV signs (Table 1) following standard procedure [19].

**Table 1:** Details of PPR serum samples collected from selected locations

Sr. No.	Location	No. of serum samples collected from goats	No. of serum samples collected from sheep	Total no. of serum samples
**1**	Fateh Jang	52	42	94
**2**	Islamabad (ICT)	109	57	166
**3**	Gilgit	44	21	65
	Total	205	120	325

The clinical signs were consistent across all affected animals that have shown mouth erosion, nasal discharge, diarrhoea, or all the above symptoms. Blood samples from these animals were randomly collected in vacutainers (BD bioscience) without any anticoagulant for antibody detection.

**Fig. 2:**
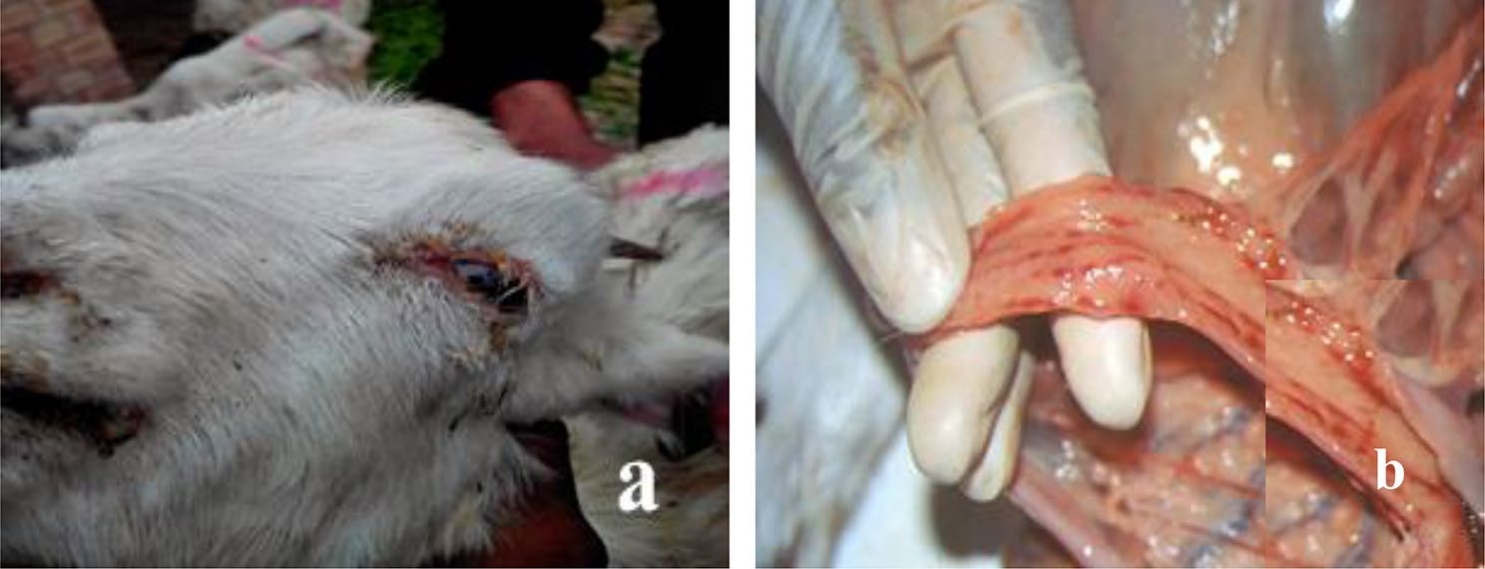
Animals presenting clinical signs of PPRV disease: **a** inflamed eye membranes; **b** evidence of “zebra striping” in the large intestine

The serum was separated by centrifuging from the blood samples (*n* = 325) and stored at 4 °C for further research process. Postmortem examinations were also performed for dead animals and six tissue samples (*n* = 06) including spleen & lungs were collected. (Fig. 2). Other clinical samples and swabs (*n* = 19) were collected from live animals in a viral transportation medium (DMEM with 2 µg/ml pen-strep antibiotics) for antigen detection purposes. Aseptic precautions were taken during the sample collection and transportation. Every sample was labelled with a unique identification number and date. These were transported to the laboratory in cool boxes and stored at -80 °C till processed further (Table 2).

**Table 2:** Sample collection from different sources for the isolation of PPRV virus

Sample sources	Total samples	Positive samples	Negative samples
Swabs	19	2	17
Tissue	6	2	4

Out of 25 clinical samples (swabs and tissues), only four samples were positive for PPRV when N-gene primer amplification was used with a positive rate of 16%, while the remaining 21 samples remained negative, as shown in Table 2.

### RNA Extraction and cDNA Synthesis

All swabs and tissue samples were subjected to RNA extraction according to the manufacturer’s instructions using a commercial RNA extraction (Ambien Pure Link RNA) Mini Kit. Reagent contamination was detected using a negative control. Extracted RNA was eluted in 50 µL nuclease-free water, which was then stored at -70 °C until used., A NanoDrop(Thermo Scientific, Wilmington, DE, USA) measured the eluted RNA concentration and a conc. of ~ 2.0 or (100ng/ul)viral RNA was used for RT-PCR. A swab was obtained from clinically healthy sheep negative for cELISA and was considered a negative control. The synthesis of the cDNA of PPRV was performed according to the manufacturer’s instructions of Revert Ad First Strand cDNA(Thermo-Scientific) [20].

### Optimization of Primers and PCR Amplification

A well-known diagnostic primer set NP3/NP4 by Couacy-Hymann was used to diagnose PPRV [21]. It was found useful in identifying, high-quality PPRV samples, like cell cultures grown viruses or any tissue samples from animals. The primer set NP3/NP4 has an N-coding gene between the positions 1232–1560 [21]. The primer set NP3/NP4, on the other hand, could be used to find the PPRV vaccine strain, but it was hard to find results when checking samples from an outbreak or with a low viral load [21]. Our investigation includes a primer set designed targeting N gene-based NP-F and NP-R from published PPRV sequences (GenBank Ac.no. KY967609 and KY967608) full genome sequences from Lahore and Faisalabad, Pakistan. These sequences were obtained from GenBank and accessed on 15th March 2018. (https://www.ncbi.nlm.nih.gov/tools/primer-blast). The BLAST tool from the NCBI database was used to search for sequenced data for gene fragments of PPRV. The CLUSTALX multiple-sequence alignment was used to target highly conserved N-coding gene’s region at nucleotide position 240–657 for further testing [22–24]. The primer sequences for these genes were NP-F (forward: 5’-AGTCACCCGGACAACTGATA-3’) and NP-R (reverse: 5’-CTTCTGCAATTCTGTTGCGG-3’) (Table 3). A few similar reports have been published [21, 25].

**Table 3:** Oligonucleotide primers set used for the PPRV detection

Primers	Sequence	Position	Amplification	Reference
NP3	5’-TCTCGGAAATCGCCTCACAGACTG-3’	1232–1560	351	Couacy-Hymann 2002,
NP4	5’-CCTCCTCCTGGTCCTCCAGAATCT-3’			
NP-F	5’-AGTCACCCGGACAACTGATA-3’	240–657	432	Designed, GenBankAc.no. KY967609andKY967608
NP-R	5’-CTTCTGCAATTCTGTTGCGG-3’			

A total of 20 µL reaction containing 4 µL of 5X PCR buffer, 0.8 µL of (10 mM) dNTPs, NP3-F (10 pmol/µl) 1.0 µL, and NP3-R (10 pmol/µl) 1.0 µL, 2 µLMgCl_2_ (100mM), 1.5 µL template cDNA, Taq polymerase (0.5 µl) and 9.2ul nuclease-free water was used. The RT-PCR cycle conditions have an initial denaturation phase at 94 °C for 10 min, 35 cycles of denaturation at 95 °C for 15 s, an annealing step at 59 °C for 1 min, extension at 72 °C for 1 min, lastly a final extension at 72 °C for 10 min and termination at 4 °C. All the tests were run at least twice.

### Virus Isolation and Propagation

The swabs and tissue samples were processed following the standard procedures [26] and inoculum was prepared for PPR virus recovery. Briefly, sub-cultures were performed using pre-cultured Vero cells (ATCC-USA). Before inoculating the Vero cell line, the medium was decanted and inoculum was dispensed (1 ml) after filtering through 0.2 μm syringe filters (Millipore). Vero cells (ATCC, USA) were grown in Dulbecco Minimum Essential Medium (100 ml of DMEM) containing10% fetal bovine serum (FBS) and 2% of pen-strep antibiotics (Corning, USA) [26]. Confluent monolayers of Vero cells were infected with previously processed and filtered tissue and swab samples. These were then incubated at 37 °C for two hours before being washed with PBS and supplemented with DMEM (maintenance media). The infected Vero cells were maintained at 37 °C for seven days and evaluated daily for any cytopathic effects (CPE). The specimen was regarded as negative when the CPE was not observed after consecutive three blind passages. The PPRV isolates were confirmed based on specific CPE and by using ‘N’ gene-based RT-PCR. The CPE, induced by *Morbillivirus*, triggered cell rounding and syncytia formation. The cells undergo three cycles of freezing and thawing, followed by centrifugation. The resultant supernatants were used for RNA extraction.

### Sequence Analysis of Selected Isolates

Sequencing was conducted and sequence data analysis was conducted following standard procedures [22, 26]. Sequence alignment was carried out using CLUSTALX software. In addition, sequenced data of Pakistani isolates were compared with other related sequences retrieved from the Gene Bank. The variations among N-gene sequences and the phylogenetic tree (analysis) of PPRV isolates used in the current study were constructed using the neighbour-joining technique in Mega-X (10.2.2). Bootstrap verification of the resulting phylogenetic tree was performed by analysis of 100 bootstraps. Given the expected similar genetic patterns, only three representative samples were randomly selected for sequencing (one from each sample collection area). Furthermore, the PCR amplicons were purified using the QIAEXII PCR purification kit (Qiagen, Venlo, Netherlands) according to the manufacturer’s instructions. Purified PCR products were sent for sequencing by Macrogen (Korea). Genius software was used to submit the sequencing to NCBI to obtain new accession numbers for our isolates from the current study.

After obtaining the N gene’s partial sequence through sequencing, different software (finch-TV software, BLASTN analysis and CLC Sequence Viewer 8) were used to analyze multiple sequence alignments to find single nucleotide polymorphisms (SNPs) (data not shown). A total of 8 sequences were selected and analyzed from various geographical locations, (including isolates from current studies), under Lineage IV of Small Ruminants Morbilliviruses (supplementary file1). Analysis of molecular variance (AMOVA) and a Nei’s distance-based cluster was also performed to examine genetic variation among populations [27], to illustrate the relationships between viral sequences from Islamabad and other regions, including previous isolates from Pakistan and other regions of world to understand its genetic evolution [28].

### Antigen Preparation

One of our sequenced PPRV local strains (ABP3-Pak), from this study, was attenuated (45th passage) in Vero cells and used to prepare IELISA antigens, as previously described [29, 30]. The PEG 6000 at 8% (w/v) in sodium chloride at 2.3% (w/v) was used to precipitate the supernatant. Following overnight incubation at 4 °C, the mixture was centrifuged at 8500 *g* for 30 min. The pellet was dissolved, one-tenth of the original volume of supernatant, in a buffer containing Tris, NaCl, and EDTA at pH 7.4 (TNE buffer) [29]. This purified antigen was stored at -80 °C until used [29]. It was important to optimize the concentration of antigen that will be used to capture antibodies but avoid nonspecific binding. The concentration of the antigen was determined using Bio Spec-Nano spectrophotometers (Shimadzu) and TCID50 quantification [30].

### Development of Indirect ELISA (IELISA)

The ELISA plates were coated with a partially purified attenuated PPR virus as the coating antigen [31]. A 96-well flat-bottom ELISA plate (NUNC Maxisorp, Hamburg, Germany) was coated with 100 ul/well of PPRV virus antigen at an optimal 1:10 dilution (10^4.8^ TCID_50_/ml) in carbonate–bicarbonate buffer (pH 9.6) and incubated overnight at 4 °C in a humid environment under constant orbital shaking conditions. Unbound antigen was removed by washing plates thrice with wash buffer, PBS-T (PBS containing 0.05% Tween-20) at pH 8.0. Every well-received 100 µL of blocking buffer (PBS-T containing 5% Bovine serum albumin) to block the remaining sites in each well. After two hours at 37 °C, the plates were again washed thrice with PBS-T wash buffer (PBS-T). Each serum sample was diluted individually (1:50) in a blocking buffer. The diluted test sample was added in 100 µL volume to individual wells in duplicates, including goat-produced positive antiserum (VNT titer > 1:4) and a negative serum (VNT titer < 1:2) from one of our previous studies (unpublished data). These positive and negative controls were implemented as previously described by Balamurugan and colleagues [31].This was followed by 2 h of humid incubation conditions at 37 °C. Anti-goat IgG HRPO-conjugated horseradish peroxidase (Abcam, UK), dissolved in 1:1000 conc. of blocking buffer, 100ul volume was added to each well and the plate was incubated for 1 h at 37 °C [32, 33].

A 100ul of substrate solution, tetra-methyl benzidine substrate (TMB), Cambridge, UK was added to each well, followed by 15 min of dark, room-temperature incubation conditions. The reaction was terminated with 100 µl of 1 M H2SO4 added to each well. ELISA readers measured the absorbance values (OD) at a wavelength of 450 nm. The mean sample/positive ratio (S/P) was calculated using OD values obtained from each tested sample. The results are expressed as percent positivity (PP) value. The PP values greater than 50% were considered positive samples [7, 34].

$$Negative{\mkern 1mu} \,control\,\left( {NCx} \right)\, = \,Mean{\mkern 1mu} \,of\,Negative\,{\mkern 1mu} Control$$

$$Positive\,{\mkern 1mu} control\left( {PCx} \right)\, = \,Mean\,{\mkern 1mu} of\,Positive{\mkern 1mu} \,Control$$

$$\frac{S}{P}value=\frac{Sample-Nc}{Pos-Nc} X100$$

### Initial Validation and Data Analysis of both Diagnostic Assays

In the indirect IELISA test, checkerboard titration was used to optimize the working dilution of antigens and antibodies. As previously described [30], the antigen and serum dilutions that presented the maximum difference between positive and negative absorbance values at 450 nm (P/N) were carefully selected. For this investigation; the reference negative serum (VNT titer < 1:2) from healthy non-vaccinated animals, and positive serum (VNT titer > 1:4) from PPRV-vaccinated animals, were tested in twofold dilutions starting from 1:2 dilutions. The antigen and serum dilutions that gave maximum difference in absorbance at 450 nm between positive and negative (P/N) were selected for testing the serum samples on larger scales. Test sera also included standard controls such as true and false positive and negative samples. According to the manufacturer’s instructions, for the cELISA kit, cutoff values of 50 and 60% were used. In contrast, a cutoff value of 50% positivity was used for the IELISA kit. The data were analyzed using a two-way contingency table using the statistical package Graph Pad Prism 5.01 [35]. Specificity and sensitivity values were determined for both I-ELISA and c-ELISA assays using the previously mentioned statistical formula [36] to compare the results. The notations presented above are explained as follows:

$$Sensitivity \left(\%\right)=a/a+c x 100$$

$$Specificity \left(\%\right)=\frac{d}{d}+b x 100$$

a = true positive (T.P.), b = false positive (F.P.), c = false negative (F.N.), d = true negative (T.N.)

### Commercial ELISA (c-ELISA) Test

PPRV antibodies were detected using a commercial c-ELISA test (ID Vet^®^ for PPR). The manipulation of the c-ELISA test was based on the manufacturer’s instructions. The ELISA microplate was read using an ELISA reader with a 450 nm filter (Bio-Rad, IMark^™^, Microplate reader) [37].

### Virus Neutralization Test (VNT)

The ability of anti-PPV antibodies to neutralize the virus was tested in Vero cells in the manner described in a previous study [29]. Briefly, the serum samples were individually incubated with 100 TCID-50 of PPRV viruses in two-fold dilutions. They were incubated at 4 C for 24 h before being poured onto the Vero cells to determine the virus infectivity rate in duplicate. These cells were then monitored daily to assess their specific cytopathic effects. Final readings were calculated on the seventh post-infection day.

### Ethical Considerations

Approval to conduct the study was received from the National Agriculture Research Center (NARC) from the Institutional Biosafety Committee (I.B.C. reference No.: NIGAB/NARC/02/05-01-2021).

## Results

### Sample Collection

During 2020–2021, out of four positive, three selected samples (one from each site) were sent for sequencing. The precise findings and details of these valuable samples are presented in Table 4, highlighting the significance of our study (Table 4).

**Table 4:** During 2020-21, the characterization of PPRV isolates from outbreak samples based on N-gene (NP-F & NP-R)

Sr. #	Sample ID	Sequencing sample ID	NCBI Accession No.	Collection date	Farm Name	Nature of sample	Area	Apparent animal status
1	Pak-GIL-1326/NARC	Local-strain (ABP3/PPRV/Islamabad/Pak/2020/1),	MW600920	15/10/2020	Hunza Farm	Goat Swab	Gilgit Baltistan	Diseased
2	Pak-FJ-1336/NARC	Local-strain (ABP3/PPRV/Islamabad/Pak/2020/2),	MW600921	5/10/2020	AlBarka Farm	Goat Swab	Fateh Jang	Diseased
3	Pak-ICT-1346/NARC	Local-strain (ABP3/PPRV/Islamabad/Pak/2020/3),	MW600922	25/10/2020	Madina Goat Farms	Goat Tissue	Islamabad	Diseased

### RNA Extraction and cDNA Synthesis

The reverse transcription-polymerase chain reaction (RT-PCR) was carried out according to the steps described in the Materials and Methods Section to check the quality and amount of cDNA synthesized from the extracted RNA. This crucial validation step ensures the accuracy of our experimental methods and the reliability of the subsequent studies’ results.

### Molecular Detection of PPRV Based on N-Genes by RT-PCR

**Fig. 3:**
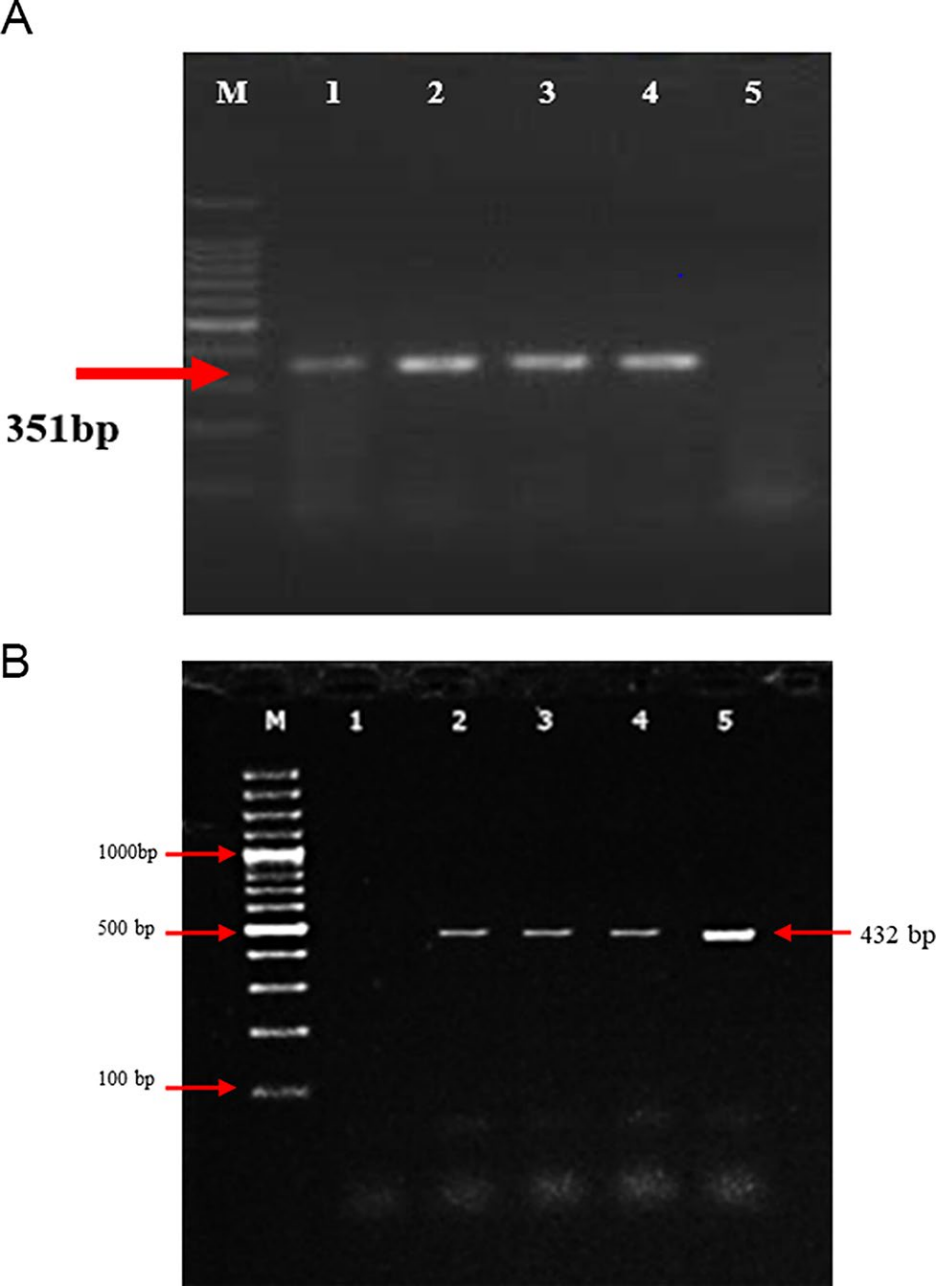
**a** Gel electrophoresis of RT-PCR products (351 bp) amplified with NP3 and NP4 primer set. Lane M: 100 bp DNA ladder (Thermo Scientific); Lane 1: positive control; Lane 2–4: swab samples obtained from clinically affected animals; Lane 5: negative control. **b** Gel electrophoresis of RT-PCR (432 bp) amplified NP3-F and NP3-R primers. Lane M: 100 bp DNA ladder (Thermo Scientific); lane 1: negative control; lane 2–4:swab samples obtained from clinically affected animals; lane 5: PPRV-positive control

For diagnosis of Peste des petits ruminant virus (PPRV) and to validate the quality and quantity of cDNA synthesized from extracted RNA, testing was carried out using the reverse transcription-polymerase chain reaction (RT-PCR) assay. Samples were carefully collected from Gilgit, Islamabad, and Fateh Jang, as illustrated in (Fig. 3a) amplification from samples of cell-cultured viral culture, using the primer pair NP3/NP4 yielded a 351 bp product. Subsequently, an RT-PCR with N-gene primers was performed, on field samples with low viral load, presenting a more specific 432 bp product (Fig. 3b). These amplifications of the N-gene indicative of PPRV, were analyzed on a 2% gel under electrophoresis at 120 V for 60 min in Tris-borate-EDTA buffer. The DNA bands were then stained with ethidium bromide and visualized under UV illumination, demonstrating clear and distinct results. Further validation was provided through the alignment of nucleic acid sequences from the PCR products with those of PPRV strains archived in GenBank. This comprehensive approach not only confirmed the presence of PPRV but also underscored the reliability of our methodologies in detecting viral genetic material.

### Phylogenetic Analysis of Indigenous Peste Des Petits Ruminants Isolates

**Fig. 4:**
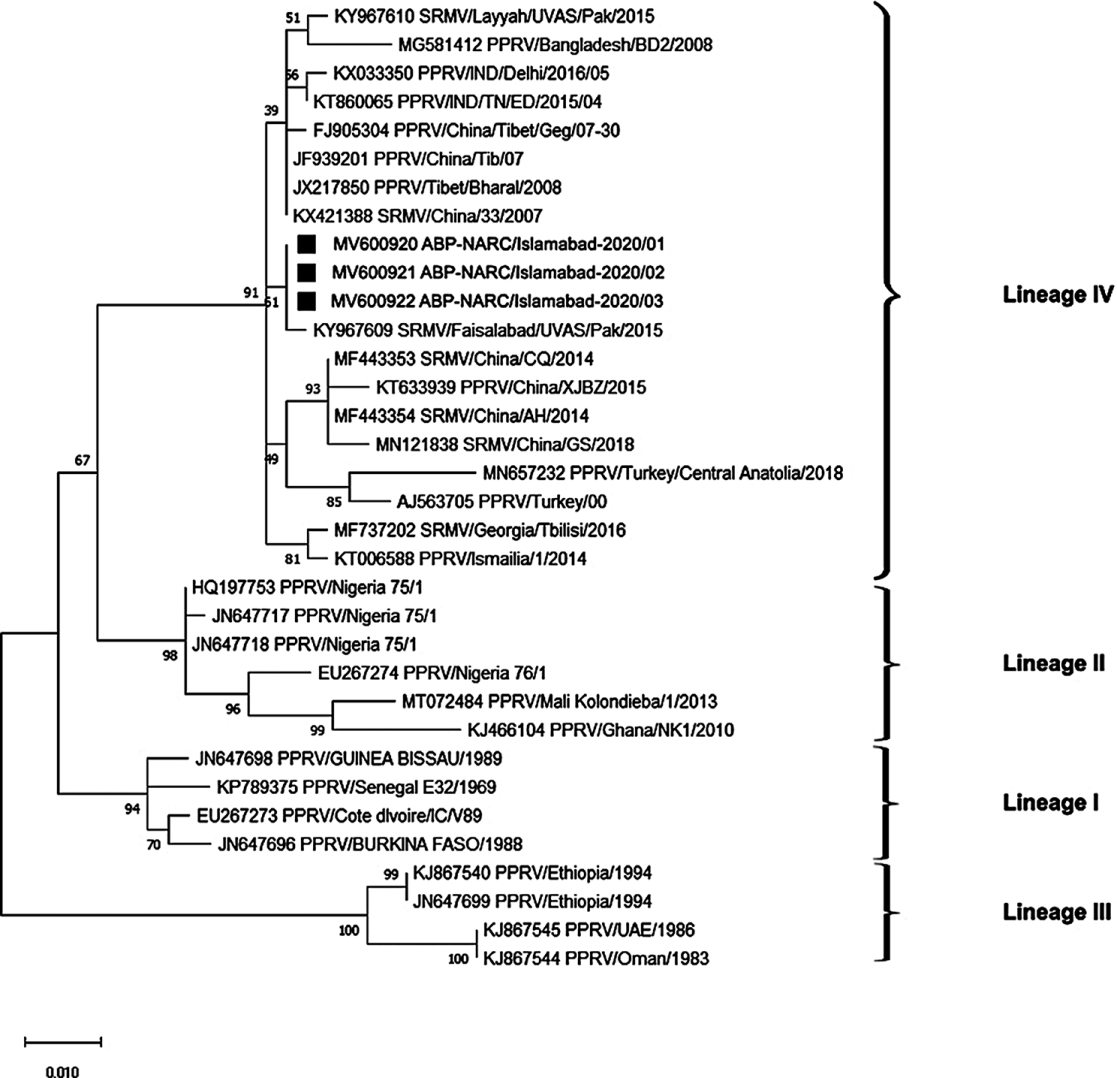
Neighbor-joining technique from N-gene sequences generated using Kimura 2 parameter model with Mega version 10.2.2, displaying 1000 replicate bootstrap results. The samples sequenced in this study are denoted by a black square (■)

Partial nucleotide sequences of the N-gene of Pakistani isolates of PPRV and isolates from other regions of Asia were available in GenBank. From these alignments, a detailed phylogenetic tree based on a 432 bp segment was constructed, revealing that Pakistani isolates robustly cluster under Lineage IV (Table: 4). These findings demonstrate a close genetic relationship with recent isolates from China, India, Bangladesh, and Turkey, enhancing our understanding of the virus’s spread and evolution in the region. The construction of this phylogenetic tree was cautiously executed using the neighbor-joining technique in Mega (10.2.2) employing the Kimura two-parameter model, as depicted in Fig. 4. To further contribute to global research efforts, all sequenced data have been deposited into GenBank and were used to construct the phylogenetic tree (Fig. 4) using the Kimura two-parameter model. All the obtained sequences were deposited in GenBank and received accession numbers MW600920, MW600921, and MW600922. The hypervariable region of the N-gene was conserved in Pakistani PPRV strains collected from Gilgit, Islamabad, and Fateh Jang. Lineage differentiation determined mainly by the N- gene, helps in understanding the worldwide movement of PPR viruses. In the present study, based on the partial N-gene sequencing method, virus strains obtained from Pakistan, China, India, Bangladesh, and Turkey were grouped under lineage IV. On phylogenetic analysis of N-gene, genetic diversity among PPRV was quite evident as represented in (Fig:4). Several sub-clusters can be observed within Lineage IV. Pakistani isolates are also segregated into two distinct sub-clusters (FIG:4) The isolate KY967610 SRMV/Layyah/UVAS/Pak/2015 can be noticed, distinct from other Pakistani isolates and clustered with an isolate from Bangladesh. It is also evident from the phylogenetic tree that PPRV isolates from the current study, formed a distinct closely related sub-cluster with a previous isolate, KY967609 SRMV/Faisalabad/UVAS/Pak/2015 from Pakistan. These findings would indicate that the N gene of Pakistan is under an evolutionary process. Isolates from Turkey showed also a distinct sub-cluster under Lineage IV PPRV. The data revealed multiple sub-clusters within Lineage IV, underscoring the dynamic genetic landscape of the virus. The fact that Pakistani isolates were split into two separate sub-clusters within this lineage shows that there are complex patterns of evolution in the area.

Furthermore, multiple sample sequence alignments were used to analyze a high rate of divergence, within genotype IV of PPRV (supplementary file S1). Analyses of molecular variance (AMOVA), were used to reveal significant genetic variation. The P-value from the AMOVA was significant as *P* < 0.05, highlighting substantial genetic differentiation among populations from Pakistan and other countries. A statistical analysis was conducted by Nei’s genetic distance calculations among isolates from Islamabad, Lahore (KY967608), Faisalabad (KY967609), India (MN369543), China (KJ720531 and FJ905304), and Bangladesh (MG581412) using allele frequencies at multiple genetic loci. This measure considers both the number of alleles shared between populations and their genetic distance. The minimum genetic distance was observed between the Lahore (KY967608) and Faisalabad (KY967609) isolates, recorded at 2.22 × 10^-16, indicating high genetic similarity. The Turkish isolate (MN657232) and both the Islamabad isolate and the Bangladesh isolate (MG581412), each with a distance of 0.875, exhibited the maximum genetic distance (Table 5).

**Table 5:**
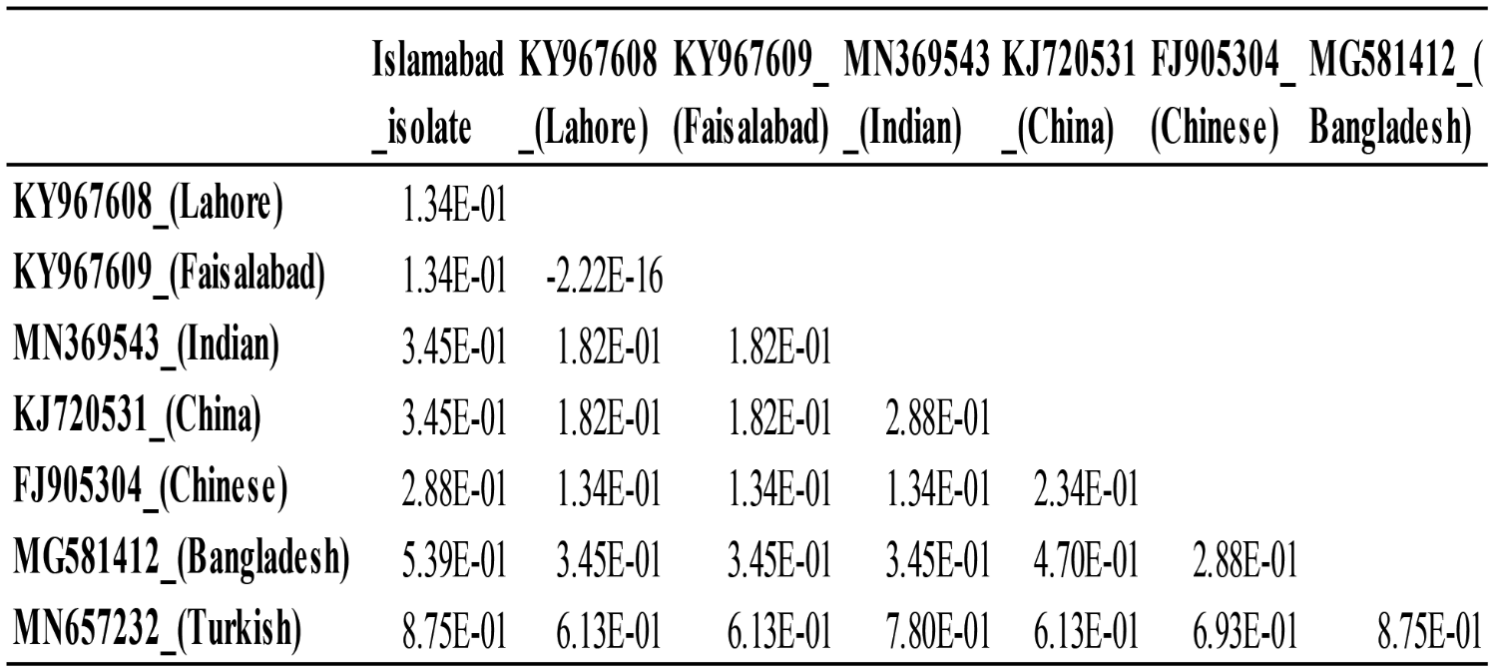
Nei’s distance table to show similarity level among isolates based on sequence data

In the present study, partial N-gene sequencing of PPRV from current isolate (Islamabad isolate) was used to analyze other lineage IV isolates of PPRV, from Pakistan (Lahore and Faisalabad), China, India, Bangladesh, and Turkey. The data revealed two distinct sub-clusters, one from Turkey and other from Bangladesh within lineage IV PPRV viruses. The Islamabad isolate exhibited more than 20 amino acid differences from the other strains studied, highlighting the dynamic genetic landscape of the virus. The division of Pakistani isolates into two distinct sub-clusters within lineage IV suggests intricate evolutionary patterns in this region (Table 5).

### Selection and Propagation of Viral Isolates

**Fig. 5:**
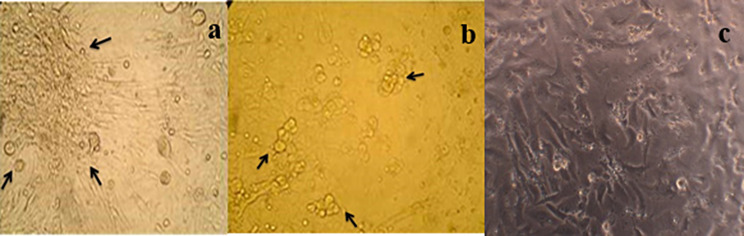
Micrograph showing cytopathic effects of PPRV in Vero cells after 14 days: **(a)** clumping of cells; **b)** rounding of cells; **(c)** mock (negative control) showing no change in morphology of monolayer

The RT-PCR test identified a total of six positive samples, demonstrating the robustness of our detection methods. In Vero cells, these samples exhibited distinct cytopathic effects. These effects were observable 14 days post-infection and included cell rounding, clumping, vacuolation, and syncytia. Three positive isolates were observed in six Vero-inoculated samples that were confirmed positive following three consecutive blind passages on Vero cells. In contrast, the mock (control) flasks showed no development of CPEs, and the monolayers remained intact, as depicted in Fig. 5. Viruses were harvested at the optimal time when 70–80% of the cells exhibited CPEs, ensuring the collection of highly active viral particles. The isolates were then subjected to a process of freeze-thawing and centrifugation. These were stored, afterwards at -80 °C, ensuring their preservation for further analysis.

### Antigen Preparation and Development of I-ELISA

**Fig. 6:**
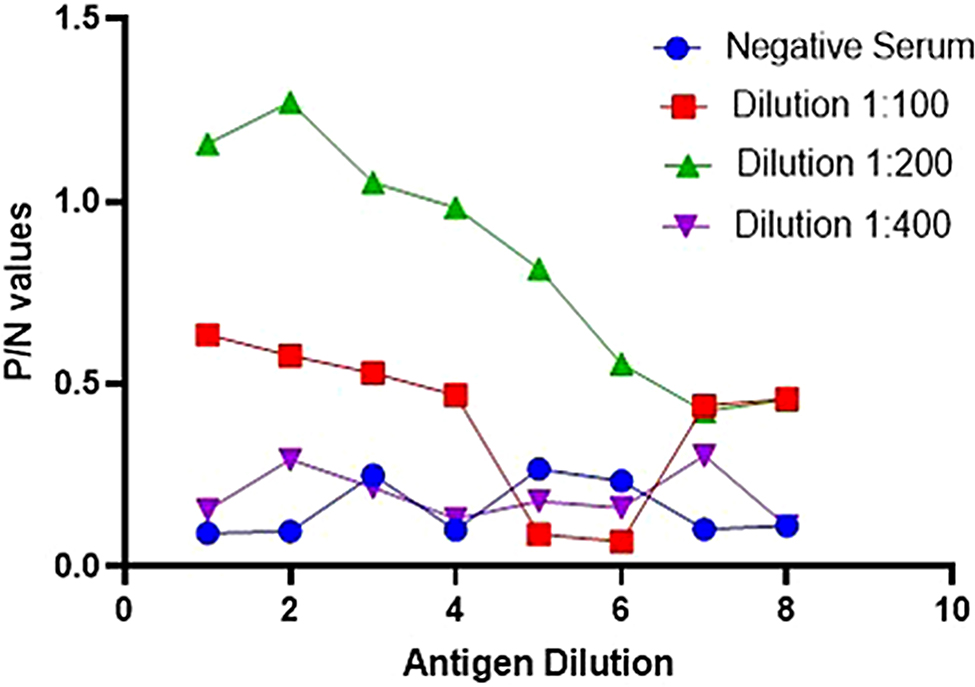
Graphical representation showing PPRV antigen (log 2) reactivity of serially diluted positive serum samples. The optimal working dilutions of antigen and antibody are 1:200 serum and 1:32 antigen

**Fig. 7:**
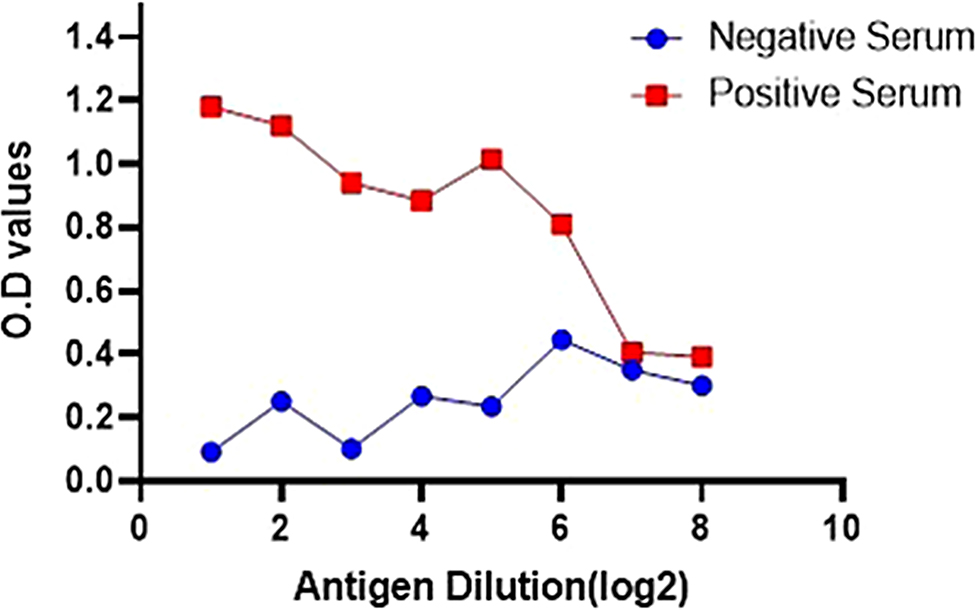
Graphical representation of positive and negative serum reactivity values at 1:200 with PPRV antigen at various dilutions. The O.D. value decreases with the decreasing concentration of the antigen

The antigen was measured by a Nanodrop spectrophotometer and found to be 455.12 (ng/µl) having 1.92 OD. The tissue culture infective dose was confirmed as 10 4.8 TCID50/ml. To ensure the accuracy of our assay, it was imperative to titrate the test serum through two-fold dilutions before selecting the optimal dilution for use. A series of antigens against different concentrations of positive serum with a virus neutralization titer (VNT titer > 4) to find the best one. Figure 6 illustrates that we achieved the maximum optical density (O.D.) at this optimal working dilution, specifically at a serum dilution of 1:200 and an antigen dilution of 1:32. Beyond this point, the OD values began to decline with decreasing antigen concentration, demonstrating a precise titration curve for both the known positive and negative sera. Our results (Fig. 7) showed a distinct titration curve for both positive and negative sera. The highest dilution of positive serum produced the highest optical density (OD), making it the best concentration for our research. Figure 7 shows that the same dilution produced the best antigen and antibody working solutions for the ELISA experiment in negative serum. Furthermore, duplicate serum samples (including positive and negative standards) were simultaneously added to three plates. No significant variation was observed in these plates. To assess the diagnostic efficacy of the IELISA assay, 325 serum samples were tested in parallel with the commercially available kit. The efficacy of IELISA was compared with a commercial kit employing all 325 serum samples.

### Relative Specificity and Sensitivity of the Developed Assay

In the current study, a two-way contingency table was used to compare how well, the indirect ELISA (IELISA) test, the competitive ELISA (cELISA), and the virus neutralization test (VNT) worked in terms of sensitivity and specificity. We analyzed a total of 325 serum samples to validate the effectiveness of the IELISA, cELISA, and VNT methods. Out of the 176 serum samples that were tested, 150 were observed to be positive via indirect IELISA and compared very well with cELISA, with a high level of specificity at 85.23% (150/176 = 0.8523) and sensitivity at 90.60% (135/149 = 0.9060) (Table 6). In comparison to a commercial kit, the specificity value was 91.43% (150/164 = 0.9143) and the sensitivity value was recorded as 84.00% (135/161 = 0.840), as shown in Table 4. Moreover, the specificity and sensitivity values obtained for IELISA were 100% and 82.14%, respectively, compared to VNT (Table 7).

**Table 6:** Relative sensitivity and specificity values of IELISA in comparison to a commercial cELISA kit

I-ELISA	Commercial kit	Negative	Total
	Positive		
**Positive**	**135(a)**	26(d)	161
**Negative**	14(b)	150(c)	164
**Total**	149	176	325

Relative specificity of assay = 150 of 176, OR 85.23%

Relative sensitivity of assay = 135 of 149, OR 90.60%

Correlation = a + c/a + b + c + dx100 = 135 + 150/325 × 100 = 87.69.7%

**Table 7:** Relative sensitivity and specificity values of IELISA in comparison to VNT

I-ELISA	VNT Assay	Negative	Total
	Positive		
**Positive**	**161(a)**	0(d)	161
**Negative**	35(b)	129(c)	164
**Total**	196	129	325

Relative specificity of assay = 129 of 129, OR 100%

Relative sensitivity of assay = 161 of 196, OR 82.14%

Correlation = a + c/a + b + c + dx100 = 161 + 129/325 × 100 = 89.23%

**Fig. 8:**
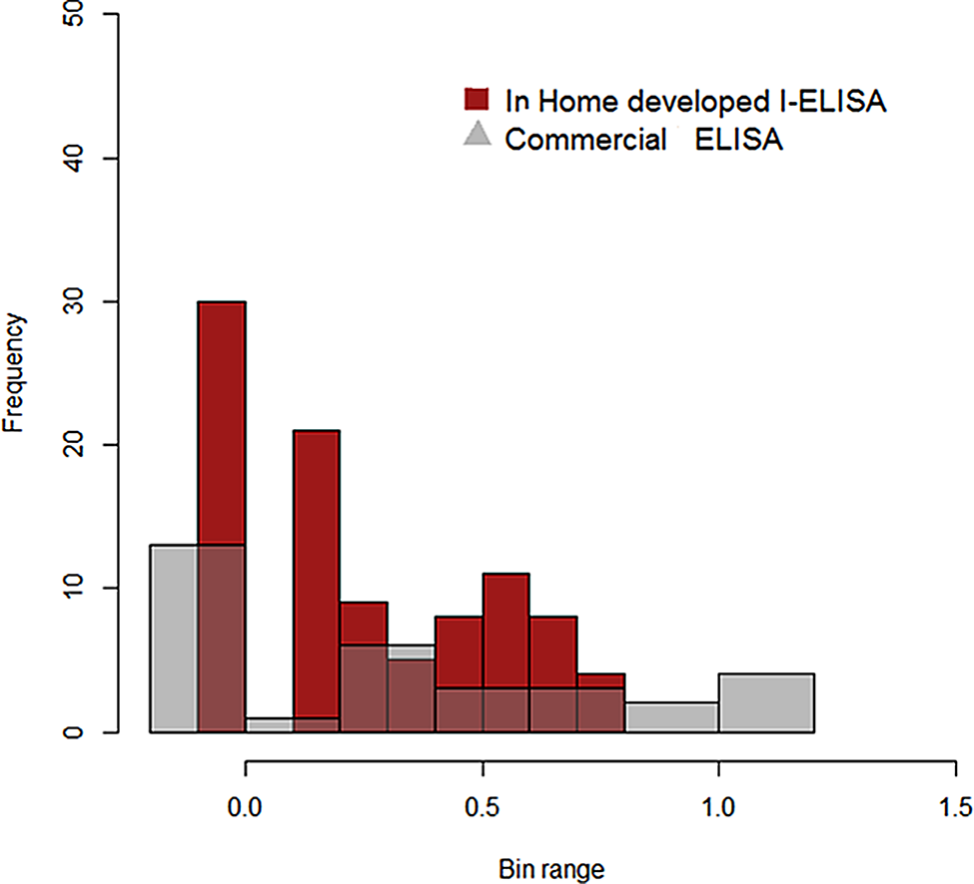
Histogram showing the distribution of percent positive values of in house developed IELISA for detecting PPRV antibodies in comparison to commercial ELISA kit

The histogram (Fig. 8) represents the sensitivity and specificity of the IELISA, determined by comparing the number of positive and negative samples detected to the known actual positive and negative samples from cELISA

Commercial kits are expensive, and component quality is poor, but an IELISA was shown to be equally specific as commercial cELISA, with few false negative findings. The application of an IELISA as, diagnostic tool for the PPR outbreak proved to be effective.

## Discussion

PPRV is a highly destructive disease that negatively impacts small ruminant productivity in Asia and other endemic regions. It adversely impacts food security and the livelihoods of poor farmers who raise sheep and goats. Un-checked trade or illegal migration across borders can spread the disease to non-endemic regions. PPRV control and eradication is crucial, like rinderpest eradication from the world. That is why, FAO and OIE launched a program called PPR Global control and eradication by 2030. The strength of any disease control program depends on using accurate and efficient diagnostic tools. These measures should involve identifying disease prevalence and symptoms, so we can minimize the spread of PPRV and reduce its impact on small ruminant productivity.

The RT-PCR-based diagnosis unveiled higher sensitivity and specificity towards field samples. The F and N genes of the virus have been targeted to confirm and differentiate the diagnosis of the PPRV virus [38]. A sequence from the Nigeria/75/1 vaccine, was used for the initial design of the NP3/NP4 primer combination [21]. But these *Morbilliviruses* are RNA viruses, known for their high degree of genetic divergence. That is due to the lack of proofreading function of polymerase that leads to nucleotide substitution errors [39]. In 2021, from Egypt Hosny and colleagues reported [40] that highlighted a significant shift in the epidemiology of PPRV, noting its expansion to new host species. It was evidenced by, camel deaths occurred in Sudan from a fatal respiratory disease that was traced back to PPRV; however, sheep and goats are the most common hosts for PPRV [41]As PPRV has been prevailing in Pakistan since1995 [42] we have expected some shifts in PPRV virus and focused our research on N-gene of *Morbillivirus*, that considered a reliable tool for the effective detection of novel strains of PPRV. This N-protein of *Morbillivirus* is known for being highly conserved and immunogenic, [43–45]. These N-gene-based primers were used in nearly every national laboratory that can conduct conventional RT-PCR, particularly in regions from Africa and Asia. A previous report suggested that primers used for PPRV diagnosis, varied in sensitivity and specificity, and new primers would require to assess the virus’s genetic diversity [7]. Another study conducted by Nafea and colleagues in 2019 [41]suggested the need for DIVA diagnostic tests to distinguish between field wild-type and vaccine PPRV strains [41]. However, inappropriate conditions like low viral load or environmental stress affect sample quality, making diagnosing difficult [7, 41].

For an effective detection of PPR viruses, it is crucial to identify conserved regions in viral genomes reported by NCBI [7]. This approach lowers the risk of false-negative results caused by primer-template disagreement [46]. In this study, newly developed NP-F and NP-R gene primers were designed from the published N- gene of the PPRV sequence (GenBank Ac. No. KY967609 and KY967608), isolated from Pakistan, which belonged to lineage IV. During the period 2020–2021, nucleotide sequencing and phylogenetic analyses revealed that the PPRV isolates obtained were clustered within lineage-IV viruses. Similar findings were reported in several previous studies [43, 47, 48]. The number of required samples and the extent of validation vary widely among labs, considering the broader validation needed for complex variant types. In our study, out of 25 (swab and tissue) samples for PPRV, only 04 samples (16%) were found positive; the rest were negative. Based on the expected genetic similarities, three samples were selected out of four positive samples, for sequence analysis. It was in line with a previous report which recommended, that to use a statistically demanding approach for sequencing validation, one should focus on specific variants or disease loci. Additionally, it stated the impracticality of validating all pathogenic variants [49]. This investigation, based on a partial sequence of N-gene presented a close relatedness with previously published sequences from Pakistan, and has allocated accession numbers (MW600920, MW600921, and MW600922) by GenBank (Fig: 4).

Notably, our findings underscore the utility of Nei’s genetic distance in revealing genetic divergence and variation among populations. The minimal distance of Islamabad isolate (2.22 × 10^-16), compared with Lahore and Faisalabad isolates suggests a high degree of genetic similarity within Pakistan. This means that their genes are very similar, which makes sense since they are so close to each other and may have shared ancestry or gene flow. The Islamabad isolate was observed to have a maximum genetic distance which is quite significant statistically i.e., 0.875 with reference to the Turkish [43] as well with Bangladesh isolates highlight the genetic distinctiveness of populations separated by greater geographical distances. The significant AMOVA results further support the presence of genetic differentiation between Pakistani populations and those from other regions. A significant *P* < 0.05 indicates that the genetic variation among the populations is statistically significant. Furthermore, this finding highlights the presence of distinct genetic structures between the Pakistani isolates and those from other countries, emphasizing the impact of geographical and possibly environmental factors on genetic differentiation. These current isolates exhibited different N-gene analysis, from earlier Pakistani isolates, which indicates that PPRVs are in a continuous process of evolution [7].

The ELISA is one of the most reliable immunological diagnostic tools currently available. A rapid ELISA would have always been a better choice over a sensitive virus neutralization test (VNT). Animal cell culture is a prerequisite for VNT but most of the laboratories do not afford it due to the lack of human resources and costly chemicals. From the current investigation, a PPRV isolate, Pak-ICT-1346/NARC was used as a coating antigen to develop an IELISA. For statistical analysis, a two-way contingency table was used to compare its sensitivity and specificity with commercially available cELISA. In this investigation, indirect IELISA performed better than cELISA and presented 150 positives out of 176 serum samples. The 90.60% sensitivity and 85.23% specificity rates are consistent with the results presented by Hosny and colleagues [40].

Commercial kits are quite expensive, and they frequently expire within a year of manufacture, resulting in very low component quality. Lower power availability and variability, along with inappropriate storage, are the main causes [50]. The indirect ELISA described here may be a useful alternative to cELISA to detect antibodies against PPRV and can be successfully used for PPR sero-epidemiological studies [31]. Developed indirect ELISA (IELISA) demonstrated 100% specificity when compared with the Virus Neutralization Test (VNT), and achieved a sensitivity of 82.14%. These findings are consistent with those reported by previous research [31], which also recorded 100% specificity and approximately 80% sensitivity for similar assays. This underscores the high sensitivity and specificity of the IELISA developed in our study, highlighting its utility and ease of use. For several reasons, cELISA is preferred over IELISA for PPRV diagnosis. Keeping in mind, the effectiveness of serological assays is always influenced by the following reasons, the disease prevalence in a specific region, the assay’s sensitivity and specificity, and the availability of required infrastructure. Additionally, it is crucial to consider the target species when selecting an assay. A previous study reported a large number of sera from atypical species, with the highest counts from African buffalo, dromedary, and Grant’s gazelle. The ID VET ELISA for African buffalo identified significantly more positives compared to the VNT and AU-PANVAC, with some results uniquely identified by the VNT only, not confirmed by the other tests [51]. Furthermore, the statistical data from the current study confirmed that our IELISA is equally effective in detecting both positive and negative samples. This capability makes it an attractive option for sero-epidemiological studies, particularly in resource-limited settings where large numbers of samples need to be tested. The IELISA offers a cost-effective alternative to more expensive cELISA kits, facilitating widespread application in field conditions and enhancing disease surveillance and control efforts [31]. Additionally, this study detailed the successful isolation of PPRV from field samples. The virus was subsequently identified by its characteristic cytopathic effect (CPE), as documented in the OIE standards of 2013 [52]. This approach further validated our methodologies in the field of virology.

Our findings highlighted the economic implications of external factors on Pakistan, particularly the exchange rate fluctuations with the U.S. dollar. Our analysis revealed that a 1% increase in the exchange rate could lead to an almost 70% surge in inflation rates and similarly impact the annual imports of Pakistan by approximately 68%. These shifts determined the vulnerability of the economy to external factors. Additionally, in terms of laboratory diagnostics, our study provides a comparison between the cost-effectiveness of the indirect ELISA (IELISA) developed here and the more expensive commercial cELISA kits [53]. Based on our cost analysis for 92 samples, where each IELISA reaction costs USD 7.81 (USD 0.21 × 92 reactions), the corresponding cost for using commercial cELISA kits ranges from USD 700 to USD 1100 for the same number of reactions. This substantial price differential underscores that the IELISA is 37% more inexpensive than its commercial counterparts for large-scale sero-epidemiological studies. Several factors were considered like the cost of reagents, labor, time, and accuracy of results. Depending on reagent availability, in-house IELISA may be inexpensive, over time. Nevertheless, the choice between using IELISA and a commercial cELISA kit may entirely depend on the laboratory’s specific requirements and available resources.

## Conclusions

The partial N-gene analysis provided a clear image of lineage-IV isolates from Pakistan. The endemic presence of peste des petits ruminant’s virus (PPRV) was monitored by successful isolation of the virus from designated areas. This evidence firmly established the sustained circulation of PPRV within these represented populations. The sequences presented offer valuable insights into the circulating PPRV strains in the country. However, beyond our findings and previously characterized sequences, there’s limited sequence data available for PPRV N genes in Pakistan. The AMOVA results revealed significant genetic differentiation between Pakistani isolates and those from other regions. Despite this limitation, our research highlights the presence of two distinct PPRV populations, underscoring the need for similar studies nationwide to accurately depict circulating viruses. Such understanding is critical for devising future control strategies. This study confirmed the effectiveness and cost-efficiency of the indirect IELISA developed for detecting PPRV-antibodies in sheep and goats. The developed IELISA offers a practical, cost-effective, and reliable method for PPRV detection, ideal for large-scale sero-epidemiological studies and control strategies, in areas where PPRV is prevalent and resources are limited.

### Electronic Supplementary Material

Below is the link to the electronic supplementary material.

Supplementary Material 1

## Data Availability

No datasets were generated or analysed during the current study.

## References

[1] Ur-Rahman A, Mirani AH, Khan M, Bukero AH, Rahman IU, Ibrahim M, Solangi NA. Peste Des Petits ruminants: a major threat to small Ruminant Health and Production. J Surv Fish Sci 2024, 15–20.

[2] Dhanasekaran S, Biswas M, Vignesh AR, Ramya R, Raj GD, Tirumurugaan KG, Subbiah E. Toll-like receptor responses to Peste Des petits ruminants virus in goats and water buffalo. PLoS ONE 2014, 9(11), e111609. 10.1371/journal.pone.0111609 PMC4219731 25369126

[3] Mdetele DP, Komba E, Seth MD, Misinzo G, Kock R, Jones BA (2021). Review of peste des petits ruminants occurrence and spread in Tanzania. Animals.

[4] Khalafalla AI. Emerging infectious diseases in camelids. Emerg Infect Dis 2017, 425–41.

[5] Dou Y, Liang Z, Prajapati M, Zhang R, Li Y, Zhang Z (2020). Expanding diversity of susceptible hosts in peste des petits ruminants virus infection and its potential mechanism beyond. Front Vet Sci.

[6] Shahriari R, Khodakaram-Tafti A, Mohammadi A (2019). Molecular characterization of Peste Des Petits ruminants virus isolated from four outbreaks occurred in southern Iran. BMC Vet Res.

[7] Mahapatra M, Neto MM, Khunti A, Njeumi F, Parida S (2021). Development and evaluation of a nested PCR for Improved Diagnosis and Genetic Analysis of Peste Des Petits ruminants Virus (PPRV) for future use in nascent PPR eradication Programme. Animals.

[8] Libeau G, Diallo A, Parida S (2014). Evolutionary genetics underlying the spread of peste des petits ruminants virus. Anim Front.

[9] Rasheed M, Akhtar T, Roohi N, Arooj N, Rasheed M, Farooq M, Yousaf M. Elucidating the genetic diversity of prevalent strains of Peste des petits ruminants virus in Gilgit-Baltistan province, Pakistan, 2020.

[10] Legnardi M, Raizman E, Beltran-Alcrudo D, Cinardi G, Robinson T, Falzon LC, Benfield CT. Peste des petits ruminants in central and Eastern Asia/West Eurasia: Epidemiological situation and status of control and eradication activities after the first phase of the PPR global eradication programme (2017–2021). *Animals* 2022, 12(16), 2030. 10.3390/ani12162030 PMC9404448 36009619

[11] Rana TK, Hassan F, Zahur AB, Ullah A, Andrabi SMH, Ali GM. The Molecular Characterization of Lineage-IV Peste Des Petits Ruminants Virus and the Development of In-House IELISA for Its Rapid Detection *Preprints* 2023. 10.1186/s12575-024-00249-y PMC11225139 38969986

[12] Kihu SM. Risk factors and socioeconomic effects associated with spread of peste des petits ruminants (PPR) in Turkana County, Kenya (Doctoral dissertation, University of Nairobi) 2014.

[13] Zhang S, Liang R, Qiu S, Zhang H, Chen Q, Niu B. Epidemic Analysis of Peste Des Petits Ruminants in India. *SSRN* 2022, 3962896. 10.1186/s12917-022-03507-x PMC9707066 36447274

[14] Zhang S, Liang R, Yang Q, Yang Y, Qiu S, Zhang H, Niu B (2022). Epidemiologic and import risk analysis of Peste Des petits ruminants between 2010 and 2018 in India. BMC Vet Res.

[15] Singh S, Transboundary. Emerging, and exotic diseases of goats. Prin Goat Dis Prev 2023, 137–54.

[16] Laxton CS, Peno C, Hahn AM, Allicock OM, Perniciaro S, Wyllie AL (2023). The potential of saliva as an accessible and sensitive sample type for the detection of respiratory pathogens and host immunity. Lan Micro.

[17] Abubakar M, Khan HA, Arshed MJ, Hussain M, Ali Q (2011). Peste Des petits ruminants (PPR): Disease appraisal with global and Pakistan perspective. Small Rumin Res.

[18] Kamel M, El-Sayed A (2019). Toward peste des petits virus (PPRV) eradication: diagnostic approaches, novel vaccines, and control strategies. Virus Res.

[19] Parida S, Selvaraj M, Gubbins S, Pope R, Banyard A, Mahapatra M (2019). Quantifying levels of peste des petits ruminants (PPR) virus in excretions from experimentally infected goats and its importance for nascent PPR eradication programme. Viruses.

[20] Chomczynski P, Sacchi NJA. Single-step method of R.N.A. isolation by acid guanidinium thiocyanate-phenol-chloroform extraction 1987, 162(1):156–9. 10.1006/abio.1987.9999 2440339

[21] Couacy-Hymann E, Roger F, Hurard C, Guillou JP, Libeau G, Diallo A (2002). Rapid and sensitive detection of peste des petits ruminants virus by a polymerase chain reaction assay. J Virol Meth.

[22] Thompson JD, Gibson T, Higgins DG (2002). Multiple sequence alignment using ClustalW and ClustalX. Curr Protoc Bioinform.

[23] Forster P, Torroni A, Renfrew C, Röhl A (2001). Phylogenetic star contraction applied to Asian and papuan mtDNA evolution. Mol Biolo Evol.

[24] Bandelt HJ, Forster P, Röhl A (1999). Median-joining networks for inferring intraspecific phylogenies. Mol Biol Evol.

[25] Sultana S, Pervin M, Sultana N, Islam MR, Khan MA (2022). H. N. A. Validation and standardization of designed N gene primer-based RT-PCR protocol for detecting Peste Des Petits ruminants virus in goats. J Adv Biotechnol Exp Ther.

[26] Kumar N, Chaubey KK, Chaudhary K, Singh SV, Sharma DK, Gupta VK, Sharma S (2013). Isolation, identification and characterization of a Peste Des Petits ruminants virus from an outbreak in Nanakpur, India. J Virol Meth.

[27] Morillo AC, Muñoz DA, Morillo Y (2023). Molecular characterization of Passiflora edulis f. Flavicarpa Degener with ISSRs markers. Braz Jour Biol.

[28] Vaz C, Nascimento M, Carriço JA, Rocher T, Francisco AP (2021). Distance-based phylogenetic inference from typing data: a unifying view. Brief Bioinf.

[29] Ahmad MUD, Burgess GW. Production and characterization of monoclonal antibodies to fowl adenoviruses. Avian Path 2001, *30*(5), 457–63. 10.1080/03079450120078635 19184933

[30] Singh RP, Sreenivasa BP, Dhar P, Roy RN, Bandyopadhyay SK (2000). Development and evaluation of a monoclonal antibody based competitive enzyme-linked immunosorbent assay for the detection of Rinderpest virus antibodies. Revue Scien Tech Int Epiz.

[31] Balamurugan V, Singh RP, Saravanan P, Sen A, Sarkar J, Sahay B, Singh RK (2007). Development of an indirect ELISA for the detection of antibodies against Peste-Des-petits-ruminants virus in small ruminants. Vet Res Commun.

[32] Spearman C (1908). The method of right and wrong cases (constant stimuli) without Gauss’s formulae. Bri Jour Psyc.

[33] Engvall E, Perlmann P (1971). Enzyme-linked immunosorbent assay (ELISA) quantitative assay of immunoglobulin. G Immunochem.

[34] Abubakar M, Qureshi M, Zahur AB, Naeem K, Khan MA, Qureshi S (2018). Field and molecular epidemiology of peste des petits ruminants in Pakistan. Pak Jour Zol.

[35] Intisar KS, Ali YH, Haj MA, Sahar MA, Shaza MM, Baraa AM, Ishag OM, Nouri YM, Taha KM, Nada EM, Ahmed AM (2017). Peste Des petits ruminants infection in domestic ruminants in Sudan. Trop Anim Heal Prod.

[36] Samad A, Awaz KB, Sarkate L (1994). Diagnosis of bovine traumatic reticulo-peritonitis I: strength of clinical signs in Predicting correct diagnosis. J Appl Anim Res.

[37] Libeau C, Prehaud R, Lancelot F, Colas L, Guerre DHL, Bishop A, Diallo (1995). Development of a competitive ELISA for detecting antibodies to the peste des petits ruminants virus using a recombinant nucleoprotein. Res Vet Sci.

[38] Kerur N, Jhala MK, Joshi CG (2008). Genetic characterization of Indian peste des petits ruminants virus (PPRV) by sequencing and phylogenetic analysis of fusion protein and nucleoprotein gene segments. Res Vet Sci.

[39] Shabbir MZ, Rahman AU, Munir M (2020). A comprehensive global perspective on phylogenomics and evolutionary dynamics of small ruminant morbillivirus. Sci Rep.

[40] Hosny W, Baheeg E, Hassanein S, Mohamed S. Preparation of a house ELISA kit for detecting Peste Des petits ruminants Virus (PPRV) antibodies. Ben Vet Med Journ 2021, 24–8.

[41] Nafea MR, Elbakry M, Shahein M, Farag GK, Abdallah F, Ali AAH (2019). Virological and molecular studies on peste des petits ruminants virus (PPRV) in small ruminants and camels in Egypt between 2017 and 2018. Adv Anim Vet Sci.

[42] Athar M, Muhammad G, Azim F, Shakoor A (1995). An outbreak of Peste Des Petits ruminants-Like Disease among goats in Punjab (Pakistan). Pak Vet Jour.

[43] Usman M, Ahsan A, Rasheed T, Farooq U, Ameen MK, Zahur AB (2019). Genetic characterization of peste des petits ruminants virus circulating in different regions of Pakistan based on nucleocapsid gene sequence. Jap Journ Vet Res.

[44] Francki RI, Fauquet CM, Knudson DL, Brown F. Classification and nomenclature of viruses: fifth report of the international committee on taxonomy of viruses. Virology division of the international union of microbiological societies. Sprin Sci Bus Med 2012.

[45] Shaila MS, Shamaki D, Forsyth MA, Diallo A, Goatley L, Kitching RP, Barrett T (1996). Geographic distribution and epidemiology of peste des petits ruminants viruses. Virus Res.

[46] Son M. *9A. y (Belbveif (Parents My Son* (Doctoral dissertation, Anand Agricultural University) 2003.

[47] Munir M, Zohari S, Saeed A, Khan Q, Abubakar M, LeBlanc N (2012). Detection and phylogenetic analysis of peste des petits ruminants virus isolated from outbreaks in Punjab, Pakistan. Trans Emer Dis.

[48] Anees M, Shabbir MZ, Muhammad K, Nazir J, Shabbir MA, Wensman JJ, Munir M (2013). Genetic analysis of peste des petits ruminants virus from Pakistan. M.C.B.M.C. Vet Res.

[49] Marshall CR, Chowdhury S, Taft RJ, Lebo MS, Buchan JG, Harrison S (2020). Best practices for the analytical validation of clinical whole-genome sequencing intended for the diagnosis of germline disease. NPJ Gen Med.

[50] McCullough KC, Sheshberadaran H, Norrby E, Obi TU, Crowther. J. R. Monoclonal antibodies against morbilliviruses 1986. 10.20506/rst.5.2.249 32917061

[51] Tully M, Batten C, Ashby M, Mahapatra M, Parekh K, Parida S, Kock R (2023). The evaluation of five serological assays in determining seroconversion to peste des petits ruminants virus in typical and atypical hosts. Sci Rep.

[52] OIE (2013). Manual of diagnostic tests and vaccines for terrestrial animals.

[53] Muñoz PM, Blasco JM, Engel B, de Miguel MJ, Marín CM, Dieste L, Mainar-Jaime RC. Assessment of performance of selected serological tests for diagnosing brucellosis in pigs. *Vet. Immun. Immunopath* 2012, 146(2), 150–158. 10.1016/j.vetimm.2012.02.012 22445082

