# Supplementary material for: Molecular Characterization of Lineage-IV Peste Des Petits Ruminants Virus and the Development of In-House Indirect Enzyme-Linked Immunosorbent Assay (IELISA) for its Rapid Detection”
Source: Biol Proced Online. 2024 Jul 5;26:22. doi: 10.1186/s12575-024-00249-y (PMC11225139; doi:10.1186/s12575-024-00249-y)
Supplement: Supplementary file 1 — Supplementary Material 1 [file 12575_2024_249_MOESM1_ESM.pdf]

Supplementary file1

Molecular characterization of lineage-IV Peste des Petits Ruminants virus and the development of in-house indirect enzyme-linked immunosorbent assay (IELISA) for its rapid detection".

Tahira Kamal [*,1,2,3,4], Saeed-ul-Hassan Khan [*,4], Fariha Hassan [3], Amir-bin-Zahoor [2], Amman Ullah [2], S. Murtaza Hassan Andrabi [2] and Ghulam Muhammad Ali [1,] Tayyaba Afsar[5], , Fohad Mabood Husain[6], Huma Shafique[7,] Suhail Razak[5]

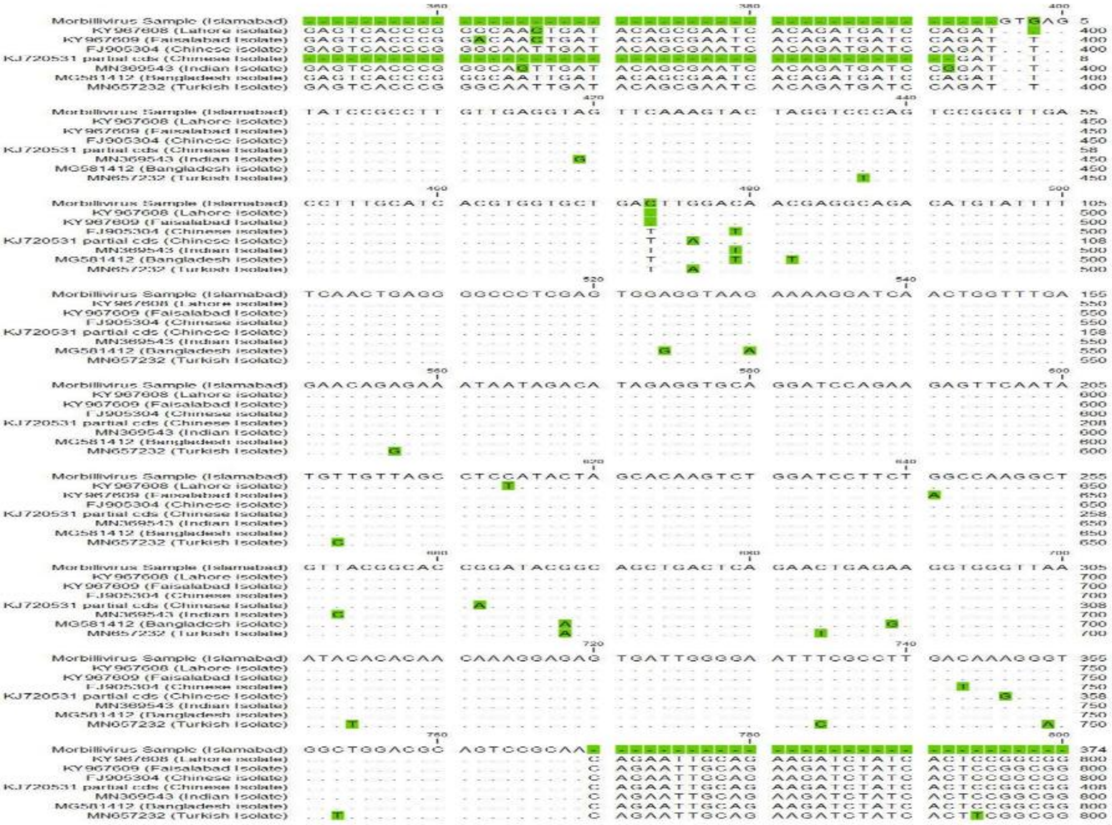

Figure 1: Multiple Sequence alignment of the sample with the sequences of lineage IV morbillivirus strains of different geographical locations i-e KY967608 (morbillivirus isolate) Lahore, KY967609 Faisalabad isolate, MN369543 Indian Isolate, KJ720531 Chinese Isolate, FJ905304 Chinese isolate, MN657232 Turkish Isolate, MG581412 Bangladesh isolate
